# Lessons learnt while integrating services for children: qualitative interviews with professional stakeholders

**DOI:** 10.1186/s12913-023-09322-w

**Published:** 2023-03-31

**Authors:** Vanessa Baxter, Ewen Speed, Vasilios Ioakimidis, Matthew Ross

**Affiliations:** School of Health & Social Care, University of Essex, Wivenhoe Park, Colchester, Essex CO4 3SQ UK

**Keywords:** Integrated care, Transformation, Children’s services

## Abstract

**Background:**

In the English NHS, integrated care is seen as an opportunity to deliver joined-up care for children and families. This paper examines the lessons learnt by professional stakeholders in the process of developing different examples of integrated models of care/frameworks for children’s services.

**Methods:**

Initial desk research was undertaken to identify different examples of integrated care models and systems/frameworks for children’s services. This identified forty-three examples in England. Of these, twelve examples were shortlisted after consultation with the senior managers within the Health and Care Partnership that had commissioned the research, and a more detailed online search for published documents was undertaken. Semi-structured qualitative interviews were then conducted with sixteen professional stakeholders in eight of these examples, ranging from one to four interviewees per example. Interviews focused on the lessons learnt from integrating and transforming services. Data were analysed using framework analysis.

**Results:**

The eight examples vary in their design but have several broad commonalities. A number of common themes and learning have emerged, of which two were identified within all eight examples: the first is about focusing on children and young people; the second is about focusing on partner engagement and collaboration and the importance of building trust and relationships between partners. A number of other important themes also emerged together with several challenges.

**Conclusions:**

A number of common factors were identified that are essential to success in integrating health and care systems. Common across all localities were being child-centric and focusing on child outcomes plus the importance of building trust, engagement and relationships with partners. The findings can help health and care system leaders transform services to ensure efficiency, improvement in services and integration.

## Background

The integration of health and social care services is claimed to be the ideal solution to increasingly complex problems in the delivery of joined-up care to the growing number of people who rely on multiple health and care services. Furthermore, it is claimed that integration enables providers and commissioners to address growing pressures of health and social care delivery created by the increasing complexity of need for services coupled with funding constraints [1, 2].

In order to try and address these challenges, various programmes to integrate health services have been developed internationally [3–6]. In England, many initiatives [7–10] have attempted to integrate health and social care systems, with varying success. From July 2022, the latest iteration of integrated care in England is the establishment of Integrated Care Systems (ICSs). These ICSs became new statutory bodies in July 2022 [11], as set out in the UK National Health Service (NHS) Long-Term Plan [12].

There are many different definitions and concepts of integrated care but NHS England (NHSE) defines it as: “Integrated care is about giving people the support they need, joined up across local councils, the NHS, and other partners. It removes traditional divisions between hospitals and family doctors, between physical and mental health, and between NHS and council services.” [13]. In developing the Rainbow Model of Integrated Care [14] it was defined as: “network[s] of multiple professionals and organisations across the health and social care system provide accessible, comprehensive and coordinated services to a population in a community.” [14 p. 3].

In practice, integrated care is difficult to achieve, especially in England where health and social care services are provided by different organisations that often have separate funding and accountability arrangements [15]. Health and care services are made up of multiple components, and the arrangements for delivering and funding them are complex [16]. The treatment and care for a single problem can require care from a range of both primary and secondary health services plus from various community-based health and care professionals. Health services are generally funded from taxes, with few charges to individuals, while social care is paid for by individuals based on means testing. Policies for integrated care have focussed on integration within the health service (for example between physical and mental health services or between GPs and hospitals) and integration between health and social care. The levels at which integration could work have been summarised by the Nuffield Trust below [17].

- **Organisational integration** focuses on coordinating structures and governance systems across organisations, such as organisational mergers, or developing contractual or cooperative arrangements.
- **Administrative or functional integration** involves joining up non-clinical support and back-office functions, for example, accounting mechanisms or sharing data and information systems across organisations.
- **Service integration** involves the coordination of different services, such as through multidisciplinary teams, single referral structures, or single clinical assessment processes.
- **Clinical integration** involves the coordination of care into a single or coherent process, either within or across professions. This could involve developing shared guidelines or protocols across boundaries of care.

The new Integrated Care Systems and their associated public sector reform are designed to bring a whole system agenda together to wrap around care provision for children and ensure that their health needs are not being separated out from all of their other needs. While the integration of services can take many forms, the focus of this paper is the integration of health and social care services for children and young people.

This study was commissioned by A Better Start Southend and the Mid & South Essex Health and Care Partnership (the latter became the Mid & South Essex ICS in July 2022). It aimed to investigate the lessons learnt by professional stakeholders while developing different examples of integrated models of care for children’s services in terms of driving up engagement, consistency and quality of existing service offers for children and families. The findings are likely to be of national relevance as organisations within the new ICS structures start to integrate services. They may also be of interest to other countries as the study has focused on the lessons learnt during the process of integrating services and the common themes from this learning could be applied to multiple types of health systems.

## Methods

The researchers undertook a comparative review of partnership and integrated approaches within a range of localities in England. The process took the form of a desk review rather than an empirical study, with researchers using the initial desk research to identify examples of integrated care models and systems/frameworks for children’s services. The aim of this review was to identify published research and reports that outlined the format of potential models of interest and/or the approaches taken to design them.

The research team needed to identify potential models of interest before they could identify relevant interviewees for that model, as there were few models of integrated care with any published research evidence available. The desk research allowed the researchers to collect information about potential examples in order to determine whether or not they truly were examples of integrated care and not merely examples of joint or partnership working.

Searches for published research, evaluations and other reports were conducted between 24th August 2021 and 29th September 2021 using the following search terms:

“Integrated care systems” OR “integrated care partnerships” OR “local care partnerships” OR “local care organisations” OR “ICS health and care partnerships” OR “child health partnerships” OR “children’s care partnerships” PLUS “models of care”, “approaches to innovation”, “structure” and “governance”.

Searches were limited to English language and published within the last five years. The databases searched for the keywords at abstract level included: Medline, PubMed and CINAHL. In addition, the key words were searched for in full using Google and Google Scholar.

This identified forty-three models or frameworks that could be of interest. The researchers agreed criteria for selecting examples for further investigation with the study commissioners, and this resulted in a shortlist of twelve examples. The key criteria for inclusion in the shortlist were that the examples should be localities that had developed strategies, frameworks or models of care where services had been or were being integrated, including examples where integrated commissioning and pooled budgets were proposed or in operation. Examples were excluded where they appeared to be ones with multi-agency working rather than integrated services or which were models of care but were not integrated across partners or which seemed to consist of a strategy without any integration of services proposed.

Contacts within four of the examples could not be identified, or the stakeholders approached did not respond to requests to take part in interviews. Eight of the twelve examples were therefore explored in more depth through semi-structured interviews with stakeholders to identify key enablers and success factors for developing and implementing them.

The eight examples varied in their design:

- Three were integrated models of care for children’s services.
- Three were frameworks for the transformation of services for children and young people.
- Two were Health and Care Partnerships for all ages with a children and young people strand within it.
- One was a Health and Care Partnership focusing on the first years of a child’s life.

The examples of interest included the three frameworks as they were being used as an “umbrella” to integrate services in those localities. While localities had integrated services in different ways, the lessons learnt while doing so proved to have many themes and experiences in common.

### Recruitment

The researchers recruited professional stakeholders in each locality partially through direct email requests (where specific individuals were identified through the desk research, or introduced to the research team by contacts within the Mid & South Essex Health and Care Partnership or via research governance staff approached for permission to interview staff within an NHS Trust) and partially through snowballing where an initial contact signposted the researchers to someone else within their organisation. The selection of potential participants was based on their involvement at a senior level in developing models of integrated care services and transformation programmes (i.e. the majority were leads for transformation or heads of services). A total of 22 senior managers/commissioners were asked to participate.

Researchers interviewed key stakeholders virtually after sending them an email invitation and participant information sheet about the study. Informed consent was gained in writing before each interview took place.

Ethics approval was granted by the University of Essex’s Research Ethics Committee 2 (Reference: ETH2021-1231). The study was conducted according to the guidelines and requirements within the University’s Code of Good Research Practice and this article conforms to the “Standards for Reporting Qualitative Research (SRQR): 21-items checklist” [18]. All participants provided written informed consent via an online consent form prior to taking part.

### Participants

Sixteen stakeholders took part in a qualitative interview, plus the researchers attended a webinar run by one of the interviewees that outlined the development of their example. Twelve of the stakeholders worked for an NHS Trust while four worked for a local authority. The roles of the interviewees are summarised below:

- Five heads of/leads for children and families transformation.
- Four executive/associate/deputy directors of children’s services.
- Three clinicians leading on a specific strand of transformation.
- One commissioning director.
- One senior contract manager.
- One operations director.
- One programme lead for patient engagement.

### Data collection

The research team developed a semi-structured interview guide focusing on the process of designing the model/approach to integrated care within each example. A number of broad areas (funding and commissioning arrangements, governance frameworks, workforce challenges, building trust and stability across partners, involvement and engagement of children and families, innovation, information systems and reporting frameworks, and any impacts seen) were pre-identified to explore in the interviews but the topic guide was very open ended allowing interviewees to highlight and discuss other areas, enablers and challenges that were not within the pre-identified areas. The average duration of interviews was approximately fifty-five minutes. The researchers subsequently sent each interviewee a copy of the findings for their model (including content from the desk research and all interviews) and asked them to check the validity and agree the content. All interviews were conducted virtually between November 2021 and February 2022 and audio-recorded with the participants’ consent.

### Data analysis

Interviews were transcribed verbatim by a professional transcriber and checked against the audio-recording by a researcher, with corrections made as appropriate. All transcripts were anonymised.

The number of interviewees per model ranged from two to four and the feedback was summarised on a model by model basis, as the study commissioners wanted to understand the lessons learnt within the context of the models developed.

The Senior Researcher, under the oversight of the Principal Investigator, developed a thematic coding framework following familiarisation with the transcripts,. The researcher produced a summary of all interviews within each locality using this format and coding framework, and then analysed all interview content to identify a number of common themes and conclusions.

### Research team

The research team comprised: the Principal Investigator, who is a qualified social worker with research interests in social work practice within an environment of change; the Senior Researcher, who has extensive experience of public sector research and evaluation gained while working for a large local authority; and the Research Assistant who is a postgraduate student.

## Results

The eight examples reviewed varied in their design but - based on analysis of the initial desk research material - had several broad commonalities with some key principles/approaches in common:

- Having a person-centred approach and a focus on the needs of the child.
- Listening to and engaging with children, young people and families.
- Driving prevention and reduction in health inequalities.
- Ensuring service design is evidence-based.
- Providing accessible and place-based services.
- Providing specialist knowledge/services within the community.
- Encouraging self-management of care.
- Collaboration or a system-wide workforce strategy.

The eight examples can be briefly summarised as follows:

1. A Community Health Partnership, including health visiting, school nursing, child and adolescent mental health services, speech and language therapy, occupational therapy and physiotherapy, and community paediatricians, plus a range of dedicated services for vulnerable children. A community interest company (a special type of limited company which exists to benefit the community rather than private shareholders) provides children’s services while a Mental Health NHS Trust provides child and adolescent mental health services.
2. Child Health GP Hubs provide vertical integration between GPs and paediatric services and horizontal integration across various community agencies (e.g. health visitors, school nurses, child and adolescent mental health services, schools, social care and children’s centres). The model has three central components: public and patient engagement; specialist outreach; and open access to specialist expertise.
3. Children and Young People Health Teams include GPs, paediatricians, psychiatrists and mental health workers, plus physical and mental health, health and social and education sectors. The model is said to be unique in the UK and across Europe in its cross-organisational, system-wide, transformative and academically rigorous approach to improving child health services. The approach comprises proactive case-finding and triage, specialist clinics, and transformative education and training for professionals working with children and young people.
4. A Children and Young People Health and Wellbeing Framework covering multiple local authorities, health organisations, educational organisations and settings and the voluntary, community and social enterprise and faith sectors. The Framework provides co-ordination and oversight of children’s health and care transformation and improvements across the area covered.
5. Three Health and Care Partnerships transforming into an ICS with collaborative working with district councils and the voluntary sector plus integration of primary and community health services. There are different models of provision within each partnership and the transformation journey is at different stages. One of the partnerships’ model is seen as unique in that it includes community paediatric provision, health visiting, school nursing and children’s centres in one single contract.
6. A Transformation Framework shaping the integration of education, health and social care services. This includes local authorities, schools, CCGs, hospitals, mental health services and the voluntary sector. The Framework sets out the key principles to plan, transform and commission services for children and young people across the locality. The core components are based on ‘proportional universalism’. The aim is to develop a graduated, responsive service offer which builds the capacity of voluntary and community resources, integrates a response to additional needs and targets resources to those most vulnerable to poor outcomes.
7. A partnership of health and care organisations that includes local authorities, Clinical Commissioning Groups, hospitals, community services, primary care, residents, mental health services and the voluntary and faith sector. The focus is on early intervention and prevention through: Universal support (maternity, health visiting, mental health and infant feeding); Universal + (specific inequalities and vulnerabilities for families); and a focus on families’ holistic needs.
8. A Children, Young People and Families Programme that includes NHS organisations, local authorities, HealthWatch, charities and the community, voluntary and social enterprise sector. The vision for the programme is: to close the gap in health and well-being outcomes for all children and young people; to give all children and young people the best start in life and the support and healthcare needed; the voice of the child and young person will be at the heart of everything the Partnership does.

A number of common themes and learning emerged. Two of these were identified within all eight examples: firstly a focus on children and young people and secondly a focus on partner engagement and collaboration and the importance of building trust and relationships between partners. A third theme that was identified as an important element for integrating services in six examples was leadership. A number of other important themes emerged in some localities but not all, including: pace of transformation; clarity of focus and vision; innovation; demonstrating impact; transformation funding; place variations; workforce development; and systems and processes. Finally, nine challenges in integrating services were also identified within examples.

All of the themes emerging from the interviews are discussed in more detail below.

### Theme 1: focusing on children and young people

Being child-centric and describing why child health is so important was seen as essential, as is ensuring that the views and experiences of children and families were embedded and put at the centre of work, by listening to and engaging with them.

#### Extract 1

*“… to really keep that profile of children and young people central to the ICS, so nobody can get away from – in any conversation – not thinking about the impact on children and young people.” (Example 6)*.

Co-production was seen as an integral part of service design/redesign by interviewees in five examples: three commission this from the voluntary sector who have expertise in this area. For another interviewee, obtaining voice data formed part of a dedicated work programme led by the voluntary sector.

#### Extract 2

*“I think you have to set the bar high with this and to actually question even the statutory organisations and what they have to do. Why are you not co-producing? Why are you not listening to your children? Why are you not designing your approach or your language or your services according to the needs of children? And I think for quite a lot of professionals that’s still very challenging, but I think we are seeing a shift in cultures and behaviours.” (Example 4)*.

### Theme 2: Partner engagement and collaboration

This was seen as a key success factor to ensure efficiency, improvement in services and integration, with a whole system approach including local authorities, health and the voluntary sector.

#### Extract 3

*“I’ve learnt a lot from working at [area] and the approach that we took in children’s services which was very much an integrated approach. And we had really good relationships with our health partners, with providers and commissioners. And I think that’s absolutely key and critical to the approach that we’re looking at.” (Example 8)*.

Interviewees in two examples suggested that it is important not to leave anybody out when designing and integrating services and to ensure that membership is representative and correct. Another suggested that it is important to get frontline workers involved right at the very beginning, rather than presenting a plan to them, but it takes much more time to do this. Engagement and liaising with key partners, both at a strategic and “shop floor” level, was essential to understand how services should be designed for a fourth example. One example acknowledged that engaging GPs is difficult but the key thing was not to try and target practices but to be flexible, start with willing ones and then grow via word of mouth.

Another example recommended initiating difficult conversations prior to Board meetings to reduce tension in meetings. Working directly with system leaders and multiple groups outside of formal reporting structures functioned to raise awareness of an issue or service offer before a business case was presented to them. This was very helpful in developing strategy for another example: subsequently feeding back to the groups providing funds about what it has been spent on and what has been achieved was helpful.

Building trust and relationships with partners was reported as being a difficult and time-consuming process but was also regarded as invaluable and essential to the progression of more integrated ways of working. Many participants reported that engagement with professionals took time and persistence and was often based on personal contact and bringing people together to discuss common issues, often through more informal channels such as coffee and chat sessions or “corridor conversations”.

#### Extract 4

*“Because we are meeting regularly we also know each other personally in that sense of having those meetings. It allows, it just helps have those difficult conversations. It’s not a magic wand, it’s hard work, it’s constant. It needs constant attention.” (Example 6)*.

Interviewees in three examples highlighted that building an understanding of the different priorities, agendas and vocabulary of each partner is important to maintain relationships while two felt that building relationships inevitably builds trust. Interviewees in four examples said that being able to have open and honest conversations, even though they are difficult, is a good way to build relationships and trust.

#### Extract 5

*“It’s really about having relationships, building trust, shared outcomes, shared approaches and then having collective agreement about how we work together going forward.” (Example 8)*.

Two interviewees reported that they felt it was important to build a culture where every part of the partnership agreed that the outcomes for children and young people need to be improved. Resilience and persistence were important in terms of changing the culture of organisations for one interviewee. Another highlighted that having somebody within the programme that can be a connector is useful, i.e. someone who has an understanding of the different agendas and how to try and make those work together to come to a solution.

#### Extract 6

*“It’s very much about what can we do as a strategic organisation to improve outcomes for children, reduce variation and embed clinical leadership.” (Example 4)*.

### Theme 3: Leadership

Interviewees in six examples reported that leadership - in terms of support and wide-ranging representativeness - is an important element, especially in building partner engagement and commitment. The excellent working relationship between the two leads from health and the local authority, and the joint accountability, were seen as a significant strength of one programme. Building relationships with senior leaders and elected members, as well as having elected members on the Board, was essential to build and maintain trust for another.

#### Extract 7

*“For example, we have a DCS and clinician co-chairing the Executive Board which, by spanning the different areas helps to deliver the outcomes needed. So, we need very much that senior leadership buy-in that children’s is a priority across the different organisations, is critical to make sure that it’s on the agenda when those conversations are happening. When strategic conversations are occurring, to have someone saying, ‘But what about children’s? What about what the children need?’” (Example 4)*.

One example includes clinical, academic and management elements within its leadership, while its Board has three co-chairs - a provider, a commissioner and a parent: this formed part of the governance arrangements for the development of the integrated model, partially in response to previous work before the integration agenda (i.e. a natural development of it) and partially to try and ensure representativeness at a leadership level for the new ways of working. Children and young people are represented at a key Executive Board in another example and were happy to challenge what was being done, or not being done. Children and/or parents form part of the working groups and sit on some programme boards within a third example.

## Other key learning points

A number of other important themes emerged across a number of localities but not all.

### Theme 4: pace of transformation

Interviewees in three examples identified that it is important to go slowly and develop a model or programme incrementally rather than all at once: many had taken a year to 18 months to be developed before implementation as genuine transformation is complicated and difficult. An interviewee in another example felt that it is important to take time to understand the complexity of children’s services and dissolve some of the barriers in order to get people to work together for a shared goal. Interviewees in another example said that starting small, or with some quick wins, helps to build momentum and buy in, develop enthusiasm and engage with a wide range of partners.

### Theme 5: clarity of focus and vision

Interviewees in four examples suggested that it is important to have a clear focus, direction and vision, while three others felt that having clarity of purpose and focus is important rather than trying to do everything at once, as is being realistic about what can be done. One interviewee said that one way of breaking down barriers between organisations and workforces had been to focus on what the model is trying to achieve – i.e. achieving child health outcomes. Another mentioned that having agreement on the key outcomes for children and a shared vision can support difficult conversations between partners. A third said that the key thing was to identify opportunities across the system to do something to add value, rather than business as usual. Making a commitment to understand issues better, and then coming back to the Board with a solution and results – to “close the loop” - worked for another example.

### Theme 6: innovation

Interviewees in five examples said that having an ethos of learning and innovation, and then being flexible - there was no one size fits all - was a key success factor for their programme. One interviewee said that a culture of innovation was created through empowering people and encouraging them to work together while also taking risks, although the latter can create tension for some partners. Three interviewees suggested that innovation must be evidence-based and/or based upon what families are saying or what frontline workers are finding, while showcasing examples of good practice was seen as being very valuable by two others.

### Theme 7: demonstrating impact

One interviewee said that demonstrating an impact from this work is important. Another said that it is important to ensure that the right metrics are measured, including experiential as well as outcome measures. Two interviewees felt that embedding a quality improvement approach is important. Another said that any newly implemented programmes are analysed for their influence on the system and how that works for each of the programme work streams.

### Theme 8: transformation funding

Having some start-up funding was seen as very useful to allow breathing space for existing services to run in parallel for a time for two examples. Interviewees in two other examples said that systems have to be realistic about funding and, if change is to be achieved, that some degree of investment is needed for transformation until it becomes business as usual.

### Theme 9: workforce development

Interviewees in two examples highlighted how developing a new “hybrid” workforce can support retention and the provision of more effective services for children and families. A third said that the workforce needs to develop skills around holistic care, requiring a training programme for physical health, mental health and social health skills. The Adversity, Trauma and Resilience work stream was a “game changer” for a fourth interviewee.

### Theme 10: place variations

Interviewees in five examples highlighted that delivery may need to vary by place, while still having a consistent approach across the locality.

### Theme 11: systems and processes

Interviewees in two examples suggested that having systems and processes in place, including admin, engagement and involvement, and data analysis, helps to maintain stability. Two others felt that it is important for staff and partners to have a firm understanding of the architecture and governance framework. It is important to have a very clear understanding of the criteria for each organisation around governance before it gets to the system governance level. However, one interviewee highlighted that the challenge is to enable robust governance while not duplicating governance processes and also moving projects speedily enough forwards.

### Theme 12: challenges

A number of challenges in integrating services were also identified. Interviewees in three examples mentioned that there are a number of challenges around Information Governance and data sharing that need to be resolved. Another interviewee said that setting up a single client record system and dataset was difficult and took much longer than expected to implement. One felt that setting out far more clearly the technicalities of joint commissioning and how to agree how resources are shared/how resources are adapted should have been done at the start. Another said that there is a need to address current workforce challenges, many of which have been exacerbated by the pandemic.

Other challenges identified were around: co-ordinating or pooling budgets within primary care and mental health; implementing national programmes within the evolving integrated care system and how to link them into the Framework and other programmes of work; how to shift business as usual services into the transformation system which can be difficult when trying to keep a focus on operational issues; overcoming the digital exclusion of families who cannot afford an internet connection; and that teams may have never met face to face, only virtually, which affected teamwork during this time and setup.

## Discussion

This study hopefully contributes to identifying professional stakeholders’ experiences of designing and integrating services for children and families, and the lessons they have learnt through doing so.

The principle of child centricity (Theme 1) is not implicit in many healthcare systems but it is seen as being extremely important for optimal child health and access to services, and therefore an essential prerequisite to the design and provision of optimal child health services [19]. This study emphasises the importance that all localities placed on putting the child at the centre of service design and transformation.

It has been suggested that bureaucratic, command-and-control approaches to management and policy are partly why efforts to integrate services fail. Instead, the fostering of an enabling environment (Theme 2) would allow a more organic collaboration between professions and organisations [20–22]. It has long been maintained that co-ordinated service delivery relies on the integration of services at multiple levels [23, 24], but the research is still evolving on how to do this in a way that would enable integration from the ground-up rather than imposing it from the top-down.

The NHS Confederation’s ICS Network has recognised that there is a risk that ICS leaders will not be able to deliver the radical changes to health and care services that the Covid-19 pandemic has demonstrated are required if they are not given sufficient time and space (Theme 4) [25]. The network identified that one of the biggest strengths of ICSs so far has been the improving of joint working between partner organisations: ninety per cent of system leaders thought that they had been able to improve joint working quite or very effectively as a result (Themes 2 and 3). The pandemic stimulated significant progress in joint working as it necessitated the quick adoption of innovative ways of working in order to adapt and respond to COVID-19 alongside an increasing demand for care (Theme 6).

Some interviewees in this study suggested that combining a clear vision and supportive and representative leadership with the building of strong and trusting relationships between professionals working in different organisations can be very effective in integrating and transforming services (Theme 5).

The key lessons learnt from the research are being used by Mid & South Essex ICS as they develop and implement their Children & Young People’s Health and Care Partnership Framework. This is a values-based partnership approach that will underpin their work as a Partnership to support children, young people and their families (Theme 5). The Framework exists not to replace the strategies and priorities held by the many organisations working across Mid and South Essex, but to make it easier to work together to build and grow the Partnership. The approach includes a number of key elements that were identified as important by interviewees: growing trusting relationships and partnerships (Theme 2); a commitment to co-production as a core belief (not an afterthought) (Theme 1); shared and courageous leadership to promote working across organisational boundaries (Theme 3); making time and space to experiment, reflect and learn (Theme 6); and the use of data and information for informed and joined up decision-making (Theme 7).

### Limitations

A potential weakness of these conclusions is the sample size of respondents recruited to the study. Many professionals will have an interest in integrating health and social care services, for children and families as well as for adults, and this study cannot claim to have sampled this range of experiences fully. The recruitment of professional participants with in-depth knowledge of integrated care led to a sample of interviewees in more senior positions and further research would benefit from including professionals of varying levels of seniority to capture a wider range of views and experiences. In addition, interviews were pragmatic and restricted in time so as to accommodate the time constraints of participants. A key area for follow up or further research would be to establish whether the themes identified are important in achieving transformation.

## Conclusions

This study found a number of common factors that are considered essential to success in integrating health and care systems. The two elements that were common across all localities were being child-centric and focusing on child outcomes plus the importance of building trust, engagement and relationships with partners. The findings of this study could help health and care system leaders work together as they try to transform services to ensure efficiency, improvement in services and integration.

## Data Availability

Due to ethical concerns, the interview transcripts cannot be made openly available. Please contact the corresponding author, Vanessa Baxter, with any queries.

## Abbreviations

GP: General Practitioner
ICS: Integrated Care systems
NHS: National Health Service (of England)
NHSE: National Health Service of England
